# Historical Sketch of the Progress of Medicine, from July to December, 1811

**Published:** 1812-01

**Authors:** 


*For the Medical and Physical Journal.*

### HISTORICAL SKETCH *of the* PROGRESS *of* MEDICINE, *from* JULY *to* DECEMBER, 1811.

"Well did Lord Bacon compare natural philosophy to a pyramid! Its basis is indeed the history of nature, of which we know a little and conjecture much: but its top is, without doubt, hid high among the clouds: it is the work which GOD worketh from the beginning to the end, infinite and inscrutable."—WATSON.

"Nos genera degustamus, non
"bibliothecas discutimus." QUINT. lib. 10.

THE sentiment of Lord Bacon, applied generally to natural philosophy by an elegant writer of the present time, has a close analogy with the science of medicine; the basis of which is, indeed, the history of nature, of which we know a little and conjecture much. But, forgetting where the true foundation of this science only lies, and intoxicated with the glitter of an imagined superstructure, medical investigations run often into the absurdities of wild, confused, and contradictory hypothesis. We may be accused of frequently repeating, that medical opinions are valuable only when founded on incontrovertible facts; and that induction in this science should be made as much from experience and experiment,* and as clearly and as faithfully, as in any other branch of natural philosophy. It would, indeed, be a dereliction of the principles we have always held, if we did not, as occasion presents, enforce what we

* By experience we mean a close and reiterated observance and notation of the phenomena of life, both in health and disease: by experiment we mean a similar observance of the effects produced on the vital principle, by articles of the materia medica, or by any curative process. Thus far it has been thought right to explain what we intend by experiment in medicine, because the word itself conveys to the sick a fear that unknown, uncertain, and perhaps fatal, means are to be employed. Far be it from us to encourage the empirical guesses and hazardous darings of bold and thoughtless ignorance. The learned physician is the only legitimate experimenter. Directed by what he already knows, and acquainted, in a great degree, with the powers and the properties of the instruments he employs, he is enabled to pursue the experimental process not only without hazard, but with benefit, to the diseased; and, by comparing and combining many results, he may establish principles and practices extensive in their influence, and, because they are true, immutable in their nature.

believe believe to be the only means of arriving at that perfectibility in the theory and practice of the medical art, of which we cannot doubt its being capable.

Feeling the value of that course of inquiry which tends to establish a knowledge of the laws of animation upon facts arising out of experiments skilfully made, observed with intelligence, and reported with fairness, we hail, with regret, however, at the pain they have occasioned the labours of Mr. Brodie. The Royal Society, by listening to the detail of experiments performed, and countenancing conclusions by permitting a report of them in its annual volume, stimulates the idle to exertion, and warms the despairing into hope. To this concurrence of motives and effects, it is, perhaps, that the public, within the last six months, owe the "Experiments on Vegetable Poisons," made with a view to ascertain the modes by which they occasion death. The design of these experiments is to explain in what manner certain substances act on the animal system, so as to occasion death independently of mechanical injury. As various substances, particularly several belonging to the vegetable kingdom, are known to produce death without any visible mechanical alteration or organic lesion, it must be an object of anxious curiosity to the physiologist and to the pathologist, to determine on which of the vital organs the poison employed exercises its primary influence, and through what medium that organ becomes affected. It was not to gratify this curiosity only, however, that Mr. Brodie instituted these experiments: they had a still more laudable view—to ascertain by what means their fatal consequences may be prevented. In a former number of this Journal was inserted the whole of this ingenious inquiry; it will here, therefore, only be proper to notice the general design, and to mark the results, as a striking feature in the progress of physiological research during the period which is treated of.

In the first class of these experiments the poisons were applied to the tongue, or alimentary canal. Alcohol, the essential oil of bitter-almonds, the juice of the leaves of aconite, an infusion of tobacco, and the empyreumatic oil of tobacco, were the substances employed: and the subjects were cats, rabbits, and dogs.

The experiments with alcohol led to a conclusion that the symptoms produced by a large quantity of spirits taken into the stomach, arise entirely from disturbance of the functions of the brain. The complete insensibility to external impressions, the dilatation of the pupils of the eyes, and the loss of motion, indicate that the functions of this organ are suspended: respiration, which is under its influence, is ill performed, and at last altogether ceases; while the head, to the action of which the brain is not directly necessary, continues to contract, circulating dark-coloured blood for some time afterwards. There is a striking analogy between the symptoms arising from spirits taken internally, and those produced by injuries of the brain. Concussion of the brain, Mr. Brodie further observes, which may be considered as the slightest degree of injury, occasions a state of mind resembling intoxication, and the resemblance is in some instances so complete, that the most accurate observer cannot form a diagnosis, except from the history of the case. Pressure on the brain, like excess of intoxication, produces loss of motion, insensibility, dilatation of the pupil; respiration becomes laboured and stertorous, is performed at long intervals, and at last altogether ceases. From circumstances which arose in the course of these experiments, in conjunction with others made with the view to ascertain the fact, Mr. Brodie concludes that the impression made on the sensorium commune is through the medium of nerves, and not by absorption of the ardent spirit. The essential oil of bitter almonds proved a most deadly poison. The animals it was employed upon were dead in a few minutes. Like alcohol, from the phenomena observed, it was believed to act on the brain through the medium of the nerves, without passing into the circulation. The juice of aconite was believed also to occasion death by destroying the functions of the brain. The experiments with the empyreumatic oil of tobacco gave results similar to those from alcohol, juice of aconite, and the essential oil of almonds: whether applied to the tongue or injected into the intestine, it did not stop the action of the heart and induce syncope, but occasioned death by destroying the functions of the brain. From experiments made with the infusion of tobacco, it appears that the deleterious principle of the genus Nicotiana, whatever it is, acts upon the principle of vitality through different organs, from accidental circumstances applying to itself only. Thus, while the oil of the Nicotiana acted directly upon the brain through the medium of the nerves, the infusion of the plant acted upon the heart. When death was occasioned by the oil, the heart continued to pulsate for some seconds after the vital principle had fled: when it was occasioned by the infusion, the heart ceased to beat even while the animal was yet alive. This is an anomaly yet unaccounted for.

In the other class of experiments the poisons were applied to wounded surfaces. Those employed were the essential oil of bitter-almonds; the juice of the leaves of aconite; the *Woorara*, a poison with which the Indians of Guiana arm the points of their arrows, and which differs essentially from the *Ticunas*; and the *Upas antiar*. For reasons before stated, it is unnecessary to go into a description of these experiments. The results were not dissimilar to those above detailed. The conclusions are, that some extinguish life by acting on and destroying the functions of the brain; that others produce their deadly effect by rendering the heart insensible to the stimulus of the blood, and thus stopping the circulation. The only practical fact that arises out of these experiments is, "that, when an animal is apparently dead from the influence of a poison, which acts simply by destroying the functions of the brain, it may, in some instances at least, be made to recover, if respiration is artificially produced, and continued for a certain length of time." In following Mr. Brodie through his investigation, we have been struck with the effect of the essential oil of almonds. The improbability, à priori, of finding such a deleterious substance in that fruit; its instantaneous and vivid effect on the vital principle, not leaving time for absorption, but travelling, like the electric fluid, along the nerves to the brain, are circumstances not less important than they are astonishing.

ing. A gentleman, who had the hardihood to apply a particle of this oil to his tongue, descr bes its effect to be like a blow on the brain. The moment it came in contact with the tongue, it was felt in the brain. To the shock succeeded instantaneously mental confusion, and the rapid approach of insensibility. The accuracy of Mr. Brodie, both in observation and detail, is remarkably borne out by the coincidence of his experiments with those made by Mr. John Pearson, of Golden-square, in the year 1806. In the Croonian Lecture on Muscular Motion, read before the Royal Society in that year, by Mı. Pearson, the discussions were chiefly limited to the subject of muscular power, with remarks on several of the circumstances under which this power is increased, diminished, or finally abolished. An investigation of this kind induced Mr. Pearson to make many experiments in order to determine the agency of heat, electricity, various chemical stimulants, and vegetable poisons, in exciting, diminishing, or destroying, the animal power in the muscular fibre.

Among the various vegetable poisons which were employed on this occasion, Mr. Pearson made use of the essential oil of almonds; for a knowledge of the powerful deleterious qualities of which he was indebted to Mr. Cooke, chemist, of Southampton-street, who also furnished him with the quantity of that liquid necessary for his experiments. This poison he found to be the most rapidly destructive of any which he employed in these inquiries; and, having ascertained, by opening the bodies of those animals that were killed by the application of a few drops of it to the tongue, that not a particle of the essential oil had passed into the œsophagus, or stomach, he concluded that the extinction of animal life, in all these instances, was occasioned by its direct effects on the nerves of the tongue, and on the sensorium commune. To this deadly poison are the custards and ratafias of the pastry-cooks indebted for their delicate flavour. It is, however, harmless enough in this dilute state. Much as the investigation of the laws of Nature by experiment may be approved, it is to be lamented that the progress of the experimenter is marked by convulsion, pain, and death. Great indeed should be the object, that is only to be approached but but through these horrors. So congenial is the feeling of an ingenious writer on this subject with our own, that we cannot forbear to gratify ourselves, and we hope our readers, by quoting from him a passage replete with humanity and good sense. "Some object of a practical nature—some prospect of discoveries likely to benefit mankind more than by a mere gratification of a learned curiosity, is almost requisite, in order completely to justify the expense of animal life—the large amount of torment as well as death, which such investigations demand. It is not easy to fancy any pursuit permitted by the laws and customs of civilised society, which does more violence to natural feelings, than a needless sporting with the sufferings of the lower animals.

We have never yet met with a man who could read the writings of the ingenious and laborious Fontana, without a comparison between the value of the illustrations which science derived from them, and the great waste of life and infliction of torture by which those acquisitions were purchased. It has been said, with some little exaggeration perhaps, that, after tormenting and destroying some thousands of innocent animals, as susceptible of pleasurable or painful sensations as himself, the Abbé arrived at the conclusion—that the poison of the viper is mortal. We own that, in reading some of Mr. Brodie's and Mr. Everard Home's experiments, we have been a little reminded of those feelings, which, we believe, are universally excited by the volumes of the Italian naturalist. Laying bare the axillary plexus—making an incision, and applying to it woorara, or oil of bitter almonds—injecting into the œsophagus so many ounces of infusion of tobacco—tying up the thoracic duct—laying open the thorax while the heart continued acting—removing the head—are all learned and delicate expressions for operations, which we shall not describe further than by saying, that they ought indeed to bring something beside sport to us, otherwise we are called upon to consider, that they are death and torment to others. Perhaps we go as far as is proper, when we allow man to use the lower creatures placed under his protection, in whatever way his physical wants, or the promotion of the solid interests of his kind, may point out."

1 The

The physiological researches of Mr. Brodie have not been confined to the preceding subject. A very interesting inquiry has been instituted to ascertain the mode in which the brain is necessary to the continuance of the action of the heart; and on the effect which the changes produced on the blood by respiration have on the heat of the animal body. Among the functions of animal life, the actions of the brain and of the heart stand foremost: one as being the source of sensibility and intelligence, the other as being the organ that propels to all parts of the animal frame what has been called the vital fluid, for the purposes of nutrition and repair, and for the supply of heat. The connection and dependence these organs have each on the other, was admitted, if not fully understood; and the supply of animal heat by the medium of the blood, consequent on changes produced in that fluid by respiration, seemed as philosophically certain as any fact in the system of modern physics. The experiments of Mr. Brodie have, however, loosened conviction on both subjects. In making experiments on animals to ascertain how far the influence of the brain is necessary to the action of the heart, he found that, when an animal was pithed,* by dividing the spinal marrow in the upper part of the neck, respiration was immediately destroyed, but the heart continued to contract circulating dark-colored blood; and that, in some instances, ten or fifteen minutes elapsed before its action had entirely ceased. He further found, that, when the head was removed, the divided blood vessels being secured by ligature, the circulation still continued, apparently unaffected by the entire separation of the brain. These facts confirmed the observations of Cruikshank *(Phil. Trans.* 1795,) and Bichat *(Réchercherches Physiologiques sur la Vie et la Mort)*, that the brain is not directly necessary to the action of the heart, and that when the functions

* Pithed, a barbarism foisted into philosophical details, by some unknown or obscure experimenter on animal life. It is now only intelligible by a periphrasis, and future generations will hardly know it as synonymous with killing. The medulla spinalis has been considered by the inventor of this term, to be similar to the pith of trees; and, when the animal is destroyed, by dividing this pith, it is said to be *pithed*.

of the brain are destroyed, the circulation ceases only in consequence of the suspension of respiration. From these facts it was fairly conjectured that the heart would continue to contract, if respiration was artificially carried on. Subsequent experiments made with this view confirmed the conjecture. In a dog, the circulation was kept on two hours and a half, after the head was separated from the body. It was observed, that, under these circumstances of artificial respiration, the secretion of urine did not go on.

In pursuing the other branch of this inquiry, that which regarded the generation and distribution of animal heat, as connected with respiration, a very unexpected result followed the experiments. We shall not confuse the subject by going into a detail of the doctrines of animal heat, which have at various periods amused, if not instructed; but shall leave Mr. Brodie's facts simply as they are stated by himself.*

In

* A result so unexpected, and so hostile to the chemical theory of animal heat, has not passed without animadversion. The following passage, extracted from a review of Mr. Brodie's paper, will shew the complexion of these animadversions. "Adverting to the origin of the present opinions respecting animal temperature, the analogy betwixt the processes of combustion and respiration, first noticed by Dr. Black, and pursuing the steps successively taken to establish by experiment what may be termed their identity, we should expect that Mr. Brodie must have paid, in his investigation, particular attention to the two leading occurrences, the disappearance of oxygen, and the formation of carbonic acid gas. That, therefore, before he pronounced respiration artificially supported, to be the same as natural respiration, and the changes consequent upon it to be equal, he would have found himself compelled to do more than merely inform us that the contractions of the heart were renewed, and that 'the blood in the arteries was seen of a florid red, and that in the veins of a dark colour.' Although we thus assert that the information most conclusive in determining the value of these experiments has been neglected, and disputing, as we certainly must under present circumstances, the inferences which have been drawn from them, we are still willing to admit that some change, similar to the proper respiratory change, did occur in the lungs of the decapitated animals, and that some caloric must have been liberated during the subsequent acquisition of the venous character by the blood. That this liberated caloric should not be sufficient to counteract the loss occasioned by throwing into the thorax a quantity of air, from 33 to 44° below the temperature of

In all the instances where he employed artificial respiration, after the separation of the head from the trunk, *he uniformly found that the animal heat decreased with greater rapidity, than when the animal was absolutely dead.* In a rabbit

---

the animal, is surely not improbable; and, until it shall be proved, by a competent experimenter, that the carbonic acid gas formed under the circumstances already stated, bears a large proportion to the quantity expired during the natural and healthy respirations of the same animal, we cannot abandon the received opinion. Whenever the trial shall be fairly made, we confidently predict that it will be unfavourable to this fresh attempt to reanimate a defunct scyon of Hunterian physiology.

"The anticipation above expressed, rests, we apprehend, upon strong grounds. When the actions dependant on life have been materially diminished, or quite destroyed, many changes of a chemical nature are diminished, or cease altogether. From the performance of motion, by means of the voluntary muscles for example, such an action takes place in the vascular system (most especially perhaps in its ultimate branches) that a greater consumption of oxygen gas occurs than whilst the body is allowed to remain at rest. This the experiments of Seguin have fully established. Another part of the caloric of temperature in animals is supplied, we believe, directly and indirectly, by the action of the secretory organs on the blood circulating through them. In the chemical change which unquestionably takes place in secretion, although a vital process, a change of temperature probably occurs; and, as in the greater number of instances of such change, it is from a lower to a higher temperature, we are led to conclude, that secretion contributes directly to the supply of animal heat. This function operates indirectly we apprehend, by increasing the disposition of the blood to combine with oxygen: an increase dependent upon the redundency of carbon, which appears to exist in the blood when it returns to the heart: part of which carbon is however, without doubt, introduced by the lacteals and the lymphatics. That secretion is prevented by tying or dividing the nerve which connects any organ with the brain, or spinal marrow, has been shewn by several physiologists. Long ago, M. Dumas observed that the secretion of gastric juice was soon diminished, and digestion suspended, by a division of the eighth pair of nerves. Mr. Brodie, as before noticed, having shewn, that in the animals employed by him no urine was secreted, we shall be justified in concluding that the liver, (an organ altering essentially the properties of the blood,) and every other gland, when the head has been completely separated, must cease to perform its proper office.

"If, in stating 'that the heat of warm-blooded animals is dependent on the chemical changes produced on the blood by the air in respiration,' Mr. Brodie had expressed the whole of 'the generally rabbit with artificial respiration, one hundred minutes after the separation of the spinal marrow, the therm. in the rectum was 90½. In another rabbit of the same size and color, one hundred minutes after it was dead, the therm. in the rectum

---

rally-received opinion,' it might be contended that the preceding observations could not have much weight. Had he, however, attentively examined the chemical opinions respecting the sources of animal heat, he must have known that, although the changes produced on the blood by respiration are held to be essential preliminaries, yet that the changes produced during the circulation are considered to be the means for actually detaching the caloric; and that, should the rate of these second changes vary in consequence of any local influence, an increased or diminished extrication of caloric, without any apparent alteration in the extent of the previous changes, would be the result. The blood might, therefore, 'appear to undergo the usual changes in the lungs,' as he asserts, and yet the supply of caloric might be generally diminished, or even partially destroyed.

"It may be proper likewise to remind Mr. Brodie, for he does not seem to have put himself in possession of the opinions which he has undertaken to controvert, that the formation of carbonic acid gas in the lungs, or in the systems of animals, which he will scarcely venture to deny, is the great support of the chemical doctrine. If, during the combustion of charcoal, oxygen gas be expended, and carbonic acid gas with free caloric produced, it is surely a legitimate conclusion, that, in the germination of seeds, the vegetation of plants, and the respiration of animals, where unquestionably oxygen gas disappears, and carbonic acid gas is produced, caloric must also be liberated. Again, if the caloric emitted in a given time by a living being, correspond, or nearly correspond, with the quantity which would be emitted were the carbon contained in the carbonic acid gas expired during that time to be converted into that gas, by combustion out of the body, the conclusion, it will be admitted, must be materially strengthened. Now, by the experiments of Dr. Crawford, it appears that, " 100 ounce measures of oxygen gas changed into carbonic acid by the respiration of a guinea-pig, shut up in a close vessel, communicated to 31℔. 7 oz. troy of water, surrounding that vessel, 17.3° of heat; while the same quantity of oxygen gas, converted into the same acid by the burning of charcoal, communicated to a like quantity of water 19.3° of heat. Very similar is the information conveyed by the experiments of Lavoisier: indeed the difference in the results is much less than (taking into consideration the great difficulty and delicacy of the experiments) we should have been led to expect. The correspondence, before spoken of, is accordingly so far established as to authorise the assertion that, ' when equal quantities of oxygen gas are converted into carbonic acid, by animal respiration and by the combustion of carbon, rectum was 93. From the whole of his experiments Mr. Brodie deduces the following conclusions:

1. The influence of the brain is not directly necessary to the action of the heart.

2. When the brain is injured or removed, the action of the heart ceases, only because respiration is under its influence; and, if under these circumstances respiration is artificially produced, the circulation will still continue.

3. When the influence of the brain is cut off, the secretion of urine appears to cease, and no heat is generated; notwithstanding the functions of respiration, and the circulation of the blood continue to be performed, and the usual change in the appearance of the blood were produced in the lungs.

4. When the air respired is colder than the natural temperature of the animal, the effect of respiration is not to generate, but to diminish, animal heat.

To this experimental inquiry into the actions of organs of

---

bon, nearly equal quantities of caloric are set free.' Should it be contended, that, although oxygen gas disappears in that process in which carbonic acid gas is evolved, yet the former gas is not consumed in the production of the latter; and therefore, that they are successive but not dependent events, we should reply, that there is too strict an agreement in the quantities of each to give any force to the objection. In the greatest number of experiments it has appeared, that, of the oxygen employed, more has been consumed than was necessary to constitute the carbonic acid gas which was collected; but, in the most recent, and, if we mistake not, the most accurate, experiments, those performed by Messrs. Allen and Pepys, the quantities exactly correspond. Possessing the knowledge of this fact, Mr. Brodie will not easily prevail upon us to believe that the loss of oxygen, and the production of carbonic acid gas, are independent of each other.

"We could with great pleasure extend these remarks much farther, for we feel interested in the defence of a doctrine advancing so many cogent claims to our support. Erelong it is probable that we may find a suitable opportunity for renewing the discussion. What we have already offered, we trust will create some hesitation in the minds of those who may be inclined to surrender to the superficial experiments of Mr. Brodie; a view of the subject of animal temperature, which, as justly represented by very acute and intelligent chemists, exhibits 'the most perfect application of chemistry to physiology, and affords even the most perfect elucidation which we yet have of any function of the living system.'"

the the first importance in the animal machine, may be subjoined an investigation of minor consideration, but one that has frequently excited the attention of physiologists. It will now hardly be doubted that fluids, taken into the stomach, pass, occasionally, from that organ into the circulation by a route shorter than by the *thoracic duct*. Many of our readers will recollect that Mr. Everard Home made attempts to ascertain the channel by which fluids thus passed, and that he believed, on certain experiments made by himself or his assistants, the spleen to be that channel. In a report of experiments given to the public within the period here treated of, he retracts this opinion; and, though these experiments go to confirm the fact of fluids passing from the stomach into the circulation by some short and direct route, they deprive the spleen of this office, and leave us still unacquainted with the uses of that viscus. Though the fact, as just observed, of fluids passing directly from the stomach into the circulation be more fully established by these experiments, no glimpse is given of the vessels that convey them.

On a former occasion Mr. Home presumed that, as a sort of appendage to the stomach, he had discovered the use of the spleen: this, however, he now candidly gives up, and, in its place, conjectures that it may furnish some secretion to be mixed with the chyle in the thoracic duct. "That the fluid contained in the cells of the spleen is a secretion there, he says, is rendered highly probable, since it is most abundant while the digestive organs are employed, and scarcely at all met with when the animal has been some time without food. The great objection to this opinion is, there being no excretory duct but the lymphatic vessels of the spleen; these however are both larger and more numerous than in any other organ; they are found in the ass to form one common trunk, which opens into a large gland on the side of the thoracic duct, just above the *receptaculum chyli*; and, when the quicksilver is made to pass through the branches of this gland, there is a trunk equally large on the opposite side, which makes an angle, and then terminates in the thoracic duct. These lymphatic vessels are equally large as the excretory ducts of any other glands, and and therefore sufficient to carry off the secretion formed in the cells of the spleen; and, where a secretion is to be carried into the thoracic duct, it would be a deviation from the general plan of the animal economy, were any but lymphatic vessels employed for that purpose. It is a strong circumstance in favour of the secretion being so conveyed, that, in one experiment, the lacteals and cells of the spleen were unusually turgid, being placed under similar circumstances, the thoracic duct being so full as not to receive their contents." The uses of such a secretion, if it actually does take place in the spleen and passes into the thoracic duct, as above asserted, is acknowledged to be at present unascertained. It is to be hoped this train will be pursued, and that, when again Mr. Home investigates the uses of the spleen, he may not be compelled to retreat from his established positions.

Though the structure and functions of animal bodies, with their healthy and morbid actions, are the principal and fundamental objects of the medical practitioner; yet are there many collateral and auxiliary branches of knowledge of considerable importance and interest to him. Natural history, botany, mineralogy, and chemistry, are so nearly allied to medicine, that they have a claim to be noticed in whatever professes to give a detail of the progress of that science. The more perfect the physician's acquaintance with created bodies, their laws and relations, the more rational will be his practice. In this view, a retrospect of circumstances in these departments of knowledge, may be said to promote the improvement of the medical art. The inquiries of Humboldt, which were noticed, at some length, in a former number of this retrospect, were directed with great intelligence to the whole circle of natural knowledge. They had a double claim to attention,—from the great skill and ability with which they were conducted, and from the remote and interesting region to which they were applied. At present, however, the inducement is to look nearer home.

Professor Davy's indefatigable application, and powers of research, in the department of science to which he is particularly devoted, have been directed to the investigation tion of "some of the combinations of oxymuriatic gas and oxygen, and in the chemical relations of those principles to inflammable bodies." In two papers in the Philosophical Transactions for this year, Mr. Davy has given a detail of his experiments, and an exposition of his conclusions, on this interesting subject; which he has, however, yet left intricate and doubtful. It was supposed that oxymuriatic gas contained oxygen in a greater proportion than it was to be found in any other body. But this ingenious chemist is inclined to believe that this *acid is a peculiar elementary substance*, more nearly resembling oxygen itself than any of its compounds, usually denominated acids. Experiments formerly made by Mr. Davy led him more than to doubt the existence of oxygen in oxymuriatic gas; the same subject is here pursued, and additional evidence is produced in support of the opinion. The difficulty of making experiments with sufficient adroitness, the accidents, nearly imperceptible, that affect the results, the general obscurity of the subject, rendered often more obscure by the new and half-understood language* in which it is explained, or attempted to be explained, we freely confess disqualify us to give an opinion whether or no oxymuriatic gas contains oxygen. The question is here, we think, plainly stated; and those of our readers who wish to go deeply into the subject, will find the elaborate details of Mr. Davy in the Phil. Trans. part i. for 1811; and at page 298, vol. xxvi. of this Journal.

In this experimental inquiry, as in many others, it has happened, that the leading object has been left undecided, while collateral facts, of no small importance, have arisen. In the present instance there are two which deserve notice. It is a general opinion that oxymuriatic gas is capable of crystal-

* "It is singular how few terms Newton invented. Whatever language he found in common use, of that he availed himself. Scarcely ever did he use a known word in a novel sense; and the language which he found ready made, he used in such a manner as to convey no inconsiderable number of discoveries the most important and the most original that mortal man has ever been permitted to make." To the inventors of new terms, and to nomenclature-mongers, this will speak with a force that should restrain them from loading science with a language that must, for a time, be little better than a jargon.

lisation lisation when exposed to a low temperature. This Mr. Davy has shewn to be erroneous. The solution of this gas in water renders that fluid more susceptible of freezing; but, if the gas be dried, and exposed in its dry state to a cold of 40° below nothing of Fahrenheit, no crystallisation takes place. So that, in fact, the congelation has been of the water mixed with the gas, while the dry gas is not susceptible of that state. An important and interesting experiment was made with the view to ascertain the manner in which oxy-muriatic gas acts in the process of bleaching. "Scheele explained the bleaching powers of the oxymuriatic gas, by supposing that it destroyed colours by combining with phlogiston. Berthollet considered it as acting by supplying oxygen. I have made an experiment, which seems to prove that the pure gas is incapable of altering vegetable colours, and that its operation in bleaching depends entirely upon its property of decomposing water, and liberating its oxygen. I filled a glass globe, containing dry powdered muriate of lime, with oxymuriatic gas. I introduced some dry paper tinged with litmus that had been just heated, into another globe containing dry muriate of lime; after some time this globe was exhausted, and then connected with the globe containing the oxymuriatic gas, and, by an appropriate set of stop-cocks, the paper was exposed to the action of the gas. No change of colour took place, and after two days there was scarcely a perceptible alteration. Some similar paper dried, introduced into gas that had not been exposed to muriate of lime, was instantly rendered white."

Very closely connected with these chemical investigations, are experimental inquiries into the state, properties, and effects, of the atmosphere. There is no doubt but this fluid, as far as it has a relation with certain localities, undergoes, at distant periods, very material changes in temperature and its effects on vegetation, and possibly on the systems of animals: these changes constitute what has been denominated, and is actually, an alteration in climate. In Great Britain, we believe, such variations in climate have been very considerable. The climate of this country now is not the same that it was in the time of Agricola, or even in that of Wil-

 liam liam of Malmsbury. We have before been called on to notice these facts by the work of Mr. Williams, on the Climate of Great Britain. That gentleman traced not only the history of those changes down to the present time, but made an attempt to account for them, particularly as they regarded, according to his assertion, the increasing humidity, cloudiness, and coldness, of the springs and summers of the late years. He was not, if we recollect aright, very successful either in his hypothesis or conclusion. At the same period was published another work on the subject of the atmosphere, of curious research and labored investigation. Many writers, especially of former times, have endeavored to ascertain the operation of atmospheric air upon the earth and its productions. It was the object of this writer, Mr. Ellis, to discover the changes induced on atmospheric air by the germination of seeds, the vegetation of plants, and the respiration of animals. The work, which Mr. Ellis published about 1806, with the object above stated, though executed with considerable care and elaboration, met with much opposition, and was controverted both in its principle and deduction. The subject, being one, however, of great interest, and intimately connected with the fashionable science, chemistry, has been again taken up by its ingenious author. The beautiful principle discovered by Dr. Priestley, and supported by Dr. Ingenhouz, and many others, that a balance in the constituent parts of the atmosphere is sustained by the opposite actions of animal and vegetable life; that is, that the oxygen destroyed or absorbed by the breathing of animals, was restored by the respiration of vegetables, seemed so consonant to the perfect wisdom of the Creator, that attacks upon it are felt like those which wound the general belief of mankind. The inference, therefore, of Mr. Ellis, that the composition of the atmosphere is acted on precisely in the same way by all the various forms of living organisation, from the animal of the most complicated structure down to the very confines of inanimate matter, is ardently resisted. The discovery of truth being, however, the great business of man in his present state, we cannot but encourage all endeavors, however hostile to a cherished opinion, which point to the accomplishment of this final destination of the human mind. The candcur, the ingenuity, and the urbanity with which this question is contended, present a fine specimen of an ingenious mind united with a philosophical temper. Among many other researches contained in Mr. Ellis's volume, comprehending some of the most intricate and abstruse operations of nature, the agency of light in promoting the coloration of plants is not the least conspicuous*. The theory of Mr. Ellis, on this subject, is entirely chemical.

"Respecting the agency of light in promoting the coloration of plants," he says, "we may observe, in the first place, that, by the chemical agency of this subtile matter, the saline compounds of plants are decomposed, and the acid and alkaline matter thus developed, combine with the colorable juices of the vegetable. In consequence of this combination, these juices are enabled to act variously on the luminous rays. When the alkali predominates, the more refrangible rays, as the violet, blue, and green, are reflected, and the other rays are extinguished; when the acid prevails, the least refrangible, or red rays, are reflected, and the others disappear; and, from intermediate admixtures of these ingredients, intermediate colors, both single and compounded, will arise. The colors, however, which these juices present to our sight, are not reflected by the colored particles, but by the opaque matter on which they are imposed, so that the colored matter transmits only, but does not reflect, light; and this light, arriving at the eye, produces an impression which conveys the sensation of the individual color. Hence too it follows, that, when light is wholly excluded, the chemical changes of the vegetable juices, which enable them to exert these actions on the colorific rays, do not take place, and consequently the green color of the leaves, which de-

* In one of the former parts of this periodical Report, we cursorily noticed the different modes of operation, at least the different and opposite effects of light, upon living and dead matter. The fact there stated, (vide Med. and Phys. Jour. vol. xxii. p. 9.) we believed, indicated that the vital principle decided the operation of the colorific process. It cannot be too often noticed, that the mathematicians of former times, and the chemists of the present day, in their endeavours to explain the operations of life, attend too little to the principle of vitality.

pends pends on the predominance of alkaline matter, and the red color of leaves and flowers, which arises from an excess of acid, are equally prevented from appearing; from the juices being unable, in this state, to decompose the solar beam, return it almost entirely unchanged to the eye, whence the objects are destitute of color, or have the appearance of whiteness. The colors of plants, therefore, depend primarily on the chemical action of light, in changing the constitution of their juices; and their juices, by their physical operation on the colorific rays, are then enabled to exhibit all their infinite variety of hues."

If the ascertained or supposed influence of the atmosphere on every production of the earth, and upon every animated being that inhabits its surface, has given to inquiries, intended to explain its operations, a preponderance in the scale of human judgment; we must not, on that account, overlook many other researches into the history of nature, of at least equal value. Among these, botany, as comprehending a detail of the culture, arrangement, and physiology, of vegetables, holds an important station: and, from its alliance with the materia medica and the practice of physic, claims a particular notice.

From the establishment of the Linnæan Society, may be dated a more correct and scientific course of botanical study in this country. Under its present enlightened president, this society has pursued its particular object with ardour and success; and has given to other branches of natural history, particularly entomology, an attention which has greatly promoted their progress. For a full support of this sentiment we might refer to the series of volumes, under the title of Transactions, which have issued from this source; but it is the tenth of this series only, as having been published within the period allotted to this retrospect, that can be noticed. This volume contains twenty-one articles, with appropriate plates. Among them, a "Botanical Description and Natural History of the Malabar Cardamom" deserves particular regard. Mr. David White, a surgeon on the Bombay establishment, has been enabled, by inquiries and examinations made on the spot, to give a very particular, and, as it should appear, correct, history of this plant. The plant producing cardamoms is a singular, if not unique, instance of one of the most valuable articles of modern luxury, being almost entirely indebted to the care of nature for its growth and perfection. Lofty hills, whose summits are ever clothed with clouds, a moist atmosphere, or copious rains for three-fourths of the year, and an exposure admitting but a limited proportion of sun-beams, are the circumstances which experience proves, are most favourable to its growth, and are the sole requisites for an abundant crop. In this country the cardamom is known principally, if not solely, as an article of the materia medica: but in Asia, we are told, it is regarded as a grateful and salubrious accessary of diet, and its employment is so universal, that it is considered to be a necessary of life. Its name, its history, culture, and preparation for the market, are minutely detailed. By Mr. White the cardamom is placed in the genus *Amomum*, but Dr. Maton, in notes annexed to this paper, proposes to place it under a new genus, to which he gives the name of *Elettaria* *.

This plant, of such universal estimation throughout Asia, and having its cultivation, if it can be so called, accompanied with such striking peculiarities, is no less remarkable, on account of the dangers to which the persons who reap it are exposed. In the situations where the cardamom grows, the green whip-snake, for the poison of which no antidote has been found, is very numerous. The season for reaping the plant is that when the insalubrity of the air is at the highest pitch: the great heats of October, succeeding to the equinoctial rains, operating upon a drenched soil, and exhaling vapours from a luxuriant undergrowth, accumulates a mass of miasmata, which, becoming intensely noxious by stagnation, occasion fevers and fluxes of a most fatal nature. A more directly painful calamity is never escaped by the cardamom reapers—that is, the numerous bites of a small variety of the *Hirudo geometra*, whose numbers are infinite and attacks incessant. The size of these leeches varies from two lines to six; and their mi-

* From Elettari, the original Malabar appellation, as given in the Hortus Malabaricus.

nuteness nuteness and gentle mode of suction seldom engage attention or excite precaution: but, true to the ancient definition, *non missura cutem, nisi plena cruoris*, they only fall off when glutted with blood, the copious flow of which indicates the authors. The simple consequence of the bite of this creature would be little felt, were it not for the abundance of a small shrubby plant, whose leaves are so acrid, or rather caustic, as to inflame by simple contact the sound skin for more than a day; and, if they touch a wound made by the leeches, frequently extended ulcerations, phagedenic in their progress, and fatal in their termination, succeed. The name of this plant is *Mouricha*, denoting in Malabar its cutting or acrimonious quality. Mr. White describes this plant, but, from the absence of flowers, was unable to ascertain its class, order, or genus.

The president of the Linnæan Society, beside his numerous pages in this volume, has gratified the admirers of Linnæus by a translation from the original Swedish, of the *Lachesis Lapponica.* The journal of Linnæus's Lapland tour, so long shut up from the curiosity of other nations, in a language little understood beyond the confines of Sweden, is happily characterised in Dr. Smith's dedication. "We here behold," says he, "not the awful preceptor of the learned world in his professorial chair, but a youthful inexperienced student, full of ardour and curiosity, such as we ourselves have been, recording his ideas and observations for his own use, not delivering them forth for the instruction of others; and, while we admire his perseverance and acuteness, we can sympathise with his embarrassments, and readily pardon his inconsiderable mistakes." Those who have contemplated the Swedish naturalist in his finished productions only, and have felt his deep, penetrating, and sublime, genius, will almost shrink from the *Lachesis Lapponica*, lest it should lower the veneration with which they have been impressed. If no man is a hero to his valet de chambre, can we expect to find the philosopher, the "awful preceptor of the learned world," in the privacy of a journal designed as a memorandum of ideas and observations? We have been much embarrassed betwixt gratitude to Dr. Smith for an entertaining entertaining book, and fears for the creator of botanical philosophy, and the true history of nature.

Botany, and the various branches of natural history, have been cultivated and improved by the spirit of research and adventure which characterises the French nation. Within the space of a few years, many illustrious examples of this have arisen. It cannot but have been observed, however, that, from the elegant Buffon downwards, a fondness for meretricious ornament has thrown an air of romance and invention over their productions. Their travellers and their naturalists have thus rendered their relations doubtful and their hypotheses visionary. But Humboldt, were he a Frenchman, and Cuvier, may redeem them from this charge. The researches of these philosophers are most extensive, and their conclusions are fairly deduced from facts. The principles which direct may not be confined to them. We have occasion now to notice a naturalist, M. Palisot de Beauvois, who is entitled to credit for pursuing the same path. His extensive acquaintance with natural history is manifested by numerous memoirs, read to the Academy of Sciences; and the facts which he has collected in remote regions we are neither inclined to doubt or dispute. The Flora of Benin and the kingdom of Oware in the gulf of Guinea, on the western shore of Africa, is what excites our attention, as being now in the course of publication. M. P. de Beauvois visited that part of Africa, of which he is now publishing the Flora, under circumstances of the most favorable kind. The son of the king of Oware had been in France some time; and he accompanied this prince in his return to his native land. The attachment of the young African enabled the French naturalist to pursue his inquiries in Oware with great facility. The result has added many facts to botanical science, and natural history in general. The number and the novelty of the observations contained in the Flora of Oware and Benin, as well as in another work in which M. Beauvois is engaged, a "History of the Insects of Africa," place him high among those whose labours have extended the boundaries of natural science. Did our design permit, many passages of much interest might be cited: circumscribed scribed as that is, we cannot but acknowledge to have been too much impressed by the following observations of Desvaux, to refuse them a place in these pages.

"Lorsque revenu au sein de sa patrie le voyageur se repose des fatigues et des dangers qu'il a courus, une de ses plus douces satisfactions est de se rappeller ce qu'il a fait, ce qu'il a vu pendant la durée de ses courses laborieuses. Le négotiant calcule les résultats avantageux du commerce dirigé vers telle ou telle region qu'il a examinée avec soin; le geomètre, le geographe, l'astronome, disposent par ordre leurs observations; le naturaliste parcourt avec une sorte de volupté les nombreuses collections qu'il a faites dans les diverses branches de l'histoire naturelle. C'est alors que les une et les autres, apres avoir étudié, comparé, s'apperçoivent qu'ils possédent des materiaux propres à étendre les bordes de la science dont ils s'occupent, s'ils les livrent a la publicité. C'est ainsi que M. Palisot de Beauvois, après avoir parcouru une partie des côtes de l'Afrique occidentale, la belle et malheureuse colonie de Saint Domingue et la Nouvelle-Angleterre, nous fait jouir maintenant de ses nombreuses reserches."

But the ever-restless and inquisitive mind of man, not content with surveying and examining whatever the surface of the earth produces, or the waters contain, penetrates into the substance of the globe itself. In no period of time has this propensity been more strongly felt, or pursued with more scientific skill, than in the present. We had occasion, in a preceding number of this report, to notice some of the wonders which this disposition has brought to light; and particularly as it regarded the remains of animals no where now existing in a living state. The labours of M. Cuvier constitute an epoch in this branch of knowledge. Endowed with intellect of a superior order, and with unwearied industry, this naturalist pursues the inquiry; and is, perhaps, as he proceeds, withdrawing the veil of mystery which hangs upon what has been called, with some boldness, the Remains of a former World. Nearly every part of the earth has concealed within its strata fossil remains of animals, either of enormous size or extraordinary form; most of which cannot

 be be referred to any existing species. M. Cuvier has demonstrated that several species of animals to which these remains belonged have entirely disappeared from among the living inhabitants of the earth. America has been abundantly prolific in these fossil bones, particularly near or on the banks of her rivers: those of the Ohio and Hudson's river have afforded them in great quantity. From the information of M. Cuvier, we are enabled to state with precision, that the bones found near the Ohio, are not those of the Siberian mammoth. "The bones from the Ohio have been long known, and were the first which convinced naturalists that certain species had become entirely extinct. The great animal to which these bones belonged, was, for a time, confounded with the mammoth of Siberia; and, though the teeth were admitted to be of a structure quite different, the name of mammoth's bones was very improperly applied to them, both in England and America. The teeth are studded with large tubercles, instead of being composed of alternate layers of bone and enamel, as in the case of the elephant and most of the graminivorous quadrupeds. The animal must, nevertheless, have had a great affinity to the elephant; yet, on account of its teeth, Cuvier refers it to a different genus, to which, because of the tubercles just mentioned, he gives the name of *Mastodonton.*" Many of our readers will remember the skeleton of the American mammoth, now the *Mastodonton,* being exhibited in London by Mr. Rembrandt Peale. A peculiarly interesting fact accompanied the discovery of this skeleton. In the midst of the bones, as they lay when discovered, there was a mass of small branches, grass, and leaves, half-bruised, among which was thought to be a species of reed now common in Virginia; the whole appearing as if it had been enveloped in a sack, which was conceived to be the stomach of the animal.

The state of our knowledge on this subject leads to the conclusion, "that the great *mastodonton*, or animal of the Ohio, was in many repects similar to the elephant, not surpassing it greatly in size, and being probably furnished with a proboscis; that the structure of its grinders refer it nevertheless to a different genus; that it probably fed, like the hippopotamus potamus and the bear, on the roots and tougher parts of vegetables; and, though, on this account, it must have frequented marshy ground, it was not made for swimming, or living in the water, and was truly a land animal; that its bones are most common in North America, and that they are fresher and better preserved than any other fossil bones." Cuvier has reckoned, in all, five species of the *mastodonton*, some of which have been found on the old continent. Humboldt, to whom we are always in debt for interesting facts in the history of nature, found one species of this animal in the kingdom of Quito, at the elevation of 1200 toises; the greatest height at which the fossil bones of quadrupeds have yet been discovered.

The nature and design of our plan does not permit a detailed view of the various memoirs of this scientific naturalist; and, if this difficulty were overcome, the result would be, perhaps, but a hurried and faint resemblance of the excellent originals.* Sweeping from one extremity of the earth to the other, he collects information from all regions; but, while employed on these extensive views of his subject, he does not leave unnoticed the facts that arise near him. The caverns in Germany, and the plaster quarries near Paris, long celebrated for the multitude of animal remains which they contain, are examined with minute care. "The former of these are found in an extensive tract of mountainous country, which begins with the Hartz, separates the valley of the Elbe from that of the Weser, proceeds eastward from those of the Rhine and the Danube, till, turning

* M. Cuvier, professor of the anatomy of animals, and secretary to the class of physical sciences in the National Institute of France, is described as adding "the enlarged views and comprehensive mind of Buffon to the turn for accurate and minute observation which distinguished his coadjutor Daubenton. To these qualities he joins that of being a fine writer; and, though, in this respect, hardly any one can rival Buffon, he has a manifest superiority in a matter of still greater importance; for, as Buffon, from a few facts, would often advance to theory with most unphilosophical precipitation, Cuvier has always proceeded with the caution of the most rigorous induction, and satisfied with deducing a few general, from a multitude of particular, truths; he seems willing to defer the last step of generalisation till all the phenomena have been examined."

 the the sources of the Elbe, it goes on to divide the vallies of the Oder and the Vistula from the plains of Hungary, or the great basin of the Danube. The extent of this mountain's chain is more than two hundred leagues. At one extremity of this line are Beauman's cave, and Scharfel's in the Hartz, described in the *Protogea* of Leibnitz. At the other extremity are the caves of Hungary, which also contain bones, and which have been known from immemorial time. Between these two extremes, are the caves of Franconia near Bayreuth, and particularly the cave of Gaylenreuth, which of all others is the richest in fossil remains. These caverns are of great extent; they are lined with stalactitical concretions, and in these concretions, near the bottom, and on the floor, are contained a vast number of bones. The bones in all of them are nearly in the same state, detached, shattered, broken, but never rubbed; a little lighter and less solid than recent bones, yet very little decomposed, containing much gelatinous matter, and not at all petrified. What is most singular is, that in all these caverns, over a distance of more than two hundred leagues, the bones are the same. Three fourths of them nearly belong to two species of bears which no longer exist. About half the remainder belong to a species of hyæna; some few belong to the tiger, or the lion; others to the wolf or dog, the fox and polecat, or to some species very nearly allied to them."

From an interesting view of Cuvier's Memoirs, published in England, we could, with great satisfaction, accumulate extracts; but we are confined by circumstances to the following citation, which contains a reflection both novel and important. "It must seem strange," says Cuvier at the termination of his 2d Memoir, "that, in a country so extensive as that which our quarries (of Paris) occupy, more than twenty leagues from east to west, there are hardly any animal remains but of one family. It can hardly be doubted, that the proportion of bones of any species has some relation to the number of that species when alive. This, therefore, indicates a condition of the animal world, corresponding very little to that which we have now before us. In the present state of the globe, the countries which make a part of of the two great continents are inhabited by animals of all the different families, each according to its latitude and the qualities of the soil. This, however, is not the case with large islands; and the condition of New Holland, in particular, may throw some light on the state of the country inhabited by the animals in our quarries."

The inquiries of M. Cuvier were particularly directed to the plaster quarries in the vicinity of Paris, and to the caverns of Germany; and his researches have served partly to establish a curious fact in the history of fossil remains: namely, that particular species of these remains are peculiar to particular strata. This fact has been illustrated still farther by an examination of the strata near London. Mr. Parkinson, whose third and final volume of the "Organic Remains of a former World" is just published, has gone very ingeniously into this subject, in "Observations on some of the Strata in the Neighbourhood of London, and on the fossil Remains contained in them." The strata which he examines are beds of blue clay—sand and gravel stratum—upper and flinty chalk—hard chalk—strata interspersed between the clay and chalk. From this examination it appears, "that exactly similar fossils are found in distant parts of the same stratum, not only where it traverses England, but where it appears again on the opposite coast: that, in strata of considerable comparative depth, fossils are found, which are not discovered in any of the superincumbent beds: that some fossils, which abound in the lower, are found in diminishing numbers through several of the superincumbent, and are entirely wanting in the uppermost, strata: that some fossils, occurring in considerable numbers in one stratum, become very rare in the adjacent portion of the next superincumbent stratum, and afterwards are lost: that fossils of one particular genus, which exist abundantly in the lower strata, and occur in several of the superincumbent ones, are not found in the three highest strata; whilst one species of that genus, but which has not been found in a fossil state, exists in our present seas: and lastly, that most of the remains which are abundant in the superior strata, are not at all found in the lower."

The individual facts, upon which this general conclusion is founded, are many of them of a nature to excite extraordinary speculations. In the stratum at Shepey are discovered fruit or ligneous seed vessels and berries, in most prodigious quantity. From this spot Mr. Francis Crow, of Feversham, has been able to select seven hundred specimens, none of which are duplicates, and very few agree with any now known. At Highgate, and also at Shepey, a resinous matter, highly inflammable, of a darkish brown colour, and yielding, on friction, a peculiar odour, has been found. The remains of an elephant were found at Shepey; and at Kew, and Walton in Essex, those of an elephant, hippopotamus, rhinoceros, and Irish fossil elk. Difficult as these positive facts are of solution, one of a negative quality is fully as unaccountable:—"*Of man, who constitutes a genus by himself, not a single decided remain has been found in a fossil state.*"

The science of geology, of which the preceding subject makes an important and interesting part, has received, particularly as it regards this country, some valuable additions by the publication of the first volume of the Geological Society's Transactions. This society, established in London in 1807, promises to elucidate, by its ability and industry, the structure and composition of the British islands, and their mineralogy, in a way that may afford gratification to the philosopher, and advantages to commerce. Among the papers which compose this volume we notice a sketch of the natural history of the Cheshire rock-salt district—observations on the physical structure of Devonshire and Cornwall—observations on the wrekin, and the great coal-field of Shropshire—a sketch of the geology of some parts of Hampshire and Dorsetshire—notice respecting the geological structure of the vicinity of Dublin—observations on some of the strata in the neighbourhood of London—and an elaborate chemical account of an aluminous chalybeate spring in the Isle of Wight. Minute as this account, by Dr. Marcet, is, as it is confined to the natural and chemical history of the spring, and does not describe its medicinal

1 qualities, qualities, we have no inducement to enlarge this cursory notice of it.

In the department of this Report particularly appropriated to the direct business of medicine, there is, we fear, not much of interest: some novelty indeed is met with, but of a nature, possibly, neither to raise full confidence nor greatly reward belief. In anatomy and surgery, the plan of Mr. Collis, of Dublin, to unite, in the schools, the two under the denomination of "Surgical Anatomy," deserves praise. In its execution an obvious method is followed, without possessing any peculiar felicity of style or manner. To the student it may be useful, but, for those who are no longer found *inter sylvas academia*, it will have no interest. For the latter, indeed, it is not intended; and the author, in equity, should not be censured for not having given what was ulterior to his design.

In the directly practical part of surgery, the profession is indebted to Dr. Hutchinson, of the Deal Hospital, for an improved method of operating in popliteal aneurism. It has been practised by him with complete success, both as to ease and security during the operation, and in its final result. (Vide this Jour. vol. 26, p. 241.) A disease of the venous system of the lower extremity, has sometimes increased to a point that has rendered even a hazardous operation desirable. The *vena saphena major*, at times, becomes so enlarged and tortuous in its course as to occasion inconvenience, abridge the use of the limb, incur the chance of serious hemorrhage, and even sphacelus. To remove some of these morbid states, and to prevent others, it has been thought expedient to tie the vessel and cut off the current of blood, the impulse of which was the immediate cause of the derangement. This operation has been successful; it has also been fatal. To obviate the hazard, it was suggested, first, we believe, by Mr. Copeland, in his Treatise on the Diseases of the Rectum and Anus, to remove the ligature immediately after it had been drawn tight about the vein. From some facts respecting arteries, stated by Dr. Jones, it was rendered probable that this would be effectual, and sufficient inflammation would be excited to produce produce adhesion of the sides of the vein, and consequent obliteration of its calibre. Several cases have been thus operated for successfully—the patient experienced little inconvenience and no hazard. If we are not misinformed, four cases have been thus treated, by Mr. Freer, of Birmingham, with entire success. If further experience should confirm this statement, it must be considered to have the claim of no inconsiderable improvement in practical surgery, generally in the ligature of vessels, and particularly in the varicose vena saphena, the operation for which, as heretofore performed, has been frequently fatal.

An appropriation of time and talent to particular departments of the medical profession, if it fails in establishing those enlarged views of nature and science which can only, perhaps, arise out of a distinct comprehension of the many analogies, connections, and dependencies, that link together the whole of created beings, gives a manual dexterity of considerable use in practice. It is upon this principle presumed, that those persons who apply to the cure of particular diseases only, have a success in the treatment of them, not common to the faculty at large. The diseases of the eye present an instance. Generally, however, it cannot be allowed that these select practitioners, whatever may be their manual dexterity, write on the subject of their peculiar destination with much correctness and precision. It would be invidious to point at particular instances, and we are no further called to pursue the subject than to Dr. Reade, who professes to have discovered a new method of cure for the *tumor sacculi lachrymalis*. In the preceding volume of our Journal, page 227, we entered fully into Dr. Reade's views, estimated his pretensions; and, to the best of our judgment, pronounced on his conclusions. If it will be for the benefit of those who are suffering under the troublesome and tedious *tumor sacculi lachrymalis*, we shall not be displeased if our opinion has been erroneous.

The history of substances possessing properties to cure diseases, as well as the theory and practice of medicine, being less under the view and controul of the senses than practical surgery, will be influenced, more or less, by the imagination; imagination: and it is from a knowledge of this bias in the mind, that we have been induced to urge the necessity of collecting plain and simple facts, instead of loading the science with hypotheses formed on conjectural data, or falsely apprehended premises. That we have not been very successful in repelling the disposition to theorise, or in promoting the only method by which true knowledge can be acquired, will be plainly perceived by our being compelled to notice details of wonderful effects, visionary conclusions, or interested designs. Nor is it impossible but this retrospect may sometimes have seemed a history of the *Corps Charlatanique*. That the present is more free from this coloring than any preceding interval, will not, perhaps, be very evident.

The disposition to empiricism was once confined to an advanced period of life. Many old ladies, and many old gentlemen, when approaching their grand climacteric, fancied they became, in a ratio agreeing with their imbecility, very successful, if not very learned, doctors. They prescribed from some favored book of receipts, or from some popular formula, for all who were weak enough to permit them. At present, this period of longing for Esculapian fame commences in much earlier life. Ladies too young for the doctorial function, officers of the army, "lights of the law, and fathers of the church," have been seized, in the full vigor, as it seemed, of life and understanding, with this strange mania. Generally, however, these people have been honest;—credulous indeed, and soon convinced on a subject they did not understand. From them society has suffered but little: but there are many, who, without believing in the efficacy of their own nostrums, make others believe; and thus collect from the public heavy contributions both of health and money. We are at a loss to know whether to place Dr. Chrestien among those who weakly believe in the efficacy of their own inventions, and therefore are honest enthusiasts,—or among those who impose *only* on the public. The *methode Jatraleptique* of this gentleman contains so much of the wonderful, and is in one part of it on a subject which has heretofore so much misled, that it certainly brings him him on the confines, if not into the region, of quackery. We are induced to entertain this opinion by his medicinal preparation of gold. An oxyde and a muriate of this metal, he asserts, has been in his hands a remedy of the most decided efficacy in syphilis, in struma in all its forms, in consumption, and in carcinoma of the uterus. It is given in very small doses, and its effects are speedily apparent.* More experience from other quarters, and more time, will be required to establish the efficacy of this questionable remedy. But, as its inventor promises to procure relief with it in some of the most dreadful and unmanageable of human afflictions, let it not be discarded without due trial.

From the oxyde of gold of M. Chrestien, to the eau medicinale of M. d'Husson, the transition is obvious. After many months have passed away, this remedy for the gout still flatters the arthritic with ease for his pains, and even with a cure for his malady. That the rich and luxurious should eagerly seize on what promises to relieve them from the visitations of a disease that "feelingly persuades them what they are," without confining them to regimen, without requiring even temperance, or in aught abridging their pleasures, gives no surprise. But that the eau medicinale will fulfil these vast promises we do not yet believe: and, though still the tide of hope and expectation flows in its favour, we see no good reason to doubt but it is rapidly

* "De toutes les maladies," say the authors of the Journal General de Medicine, "la syphilis est sans contredit celle que la médicine combat avec le plus d'efficacité, depuis qu'on a su regulariser l'administration des diverses préparations mercurielles ; et s'il existe en medicine un spécifique, c'est certainment le mercure contre les maladies vénériennes ; il suffit seulement de proportioner l'activité de ce moyen a la gravité de la maladie, à la susceptibilité de l'individu, etc. ; et la découverte d'un nouveau remède contre ce genre d'affections n'inspireroit peut-être qu'un mediocre interet si cette découverte se bornoit là ; mais les reserches de M. Chrestien, ont poussé bien plus loin l'efficacité et les propriétes du muriate d'or. Ainsi, il a combattu avec advantage, par ce moyen, les *phthisies scrophuleuses, les phthisies tuberculeuses, les squirrhés de la matrice, les dartres, le goître et les scrophules.* Enfin il propose l'usage de ce moyen contre la plupart des maladies du systême lymphatique. Tome xxxix. p. 421.

verging verging to that gulph from whence none of its brethren have returned. Fully as we are convinced that this remedy possesses no specific power over the gout, we are not displeased with observing gentlemen of sufficient skill undertaking to discover, experimentally, its composition, because we are also convinced that it possesses great powers, and that those powers, when the substance which affords them is known, may be turned to some reasonable account in the practice of medicine. The great question, however, is, whether the eau medicinale be not some obsolete remedy vamped up anew, or is really formed with some material hitherto unnoticed by physicians. In either case analysis will not be misemployed. If it is decided to be of the former kind, the public will, as far as the public is capable, be emancipated from a juggling imposition. If it is discovered to be of the latter, an article will be added to the materia medica, which, by judicious and honest management, may be rendered serviceable to mankind. As far as inquiry has hitherto gone, the evidence proves that no discovery of a new medicinal substance has been made by this mysterious M. d'Husson. The investigation of Mr. James Moore, (vide Jour. 26, p. 224,) which has been ably and candidly conducted, almost proves that the eau medicinale is a composition of veratrum *(white hellebore)* and opium.*

But we turn from these minor considerations, the doubts and the difficulties of analytical inquiry, the jostling of quacks, and the credulity of those who would be made whole by legerdemain, to a subject of the highest importance to the human race. Vaccination, which we once thought was taking a Charlatanic tinge, (the scientific discoverer will pardon the observation,) is fast arriving, in despight of the follies of its friends, and, the policy of its enemies, at a decided character; and, in the opinion of those whose opinion is most valuable, is firmly establishing itself as a security against the attacks of variola. Both its successes and

* See also an ingenious paper on this subject by Mr. Hume, to whom we have been much indebted, at various times, for acute and masterly observations, in his particular department of the profession, Journal xxvi. 185.

 its its failures lead to the same conclusion. In every country where vaccination has been employed, it is an incontrovertible fact, that deaths by small-pox have been diminished; and that, in some countries, that disease is hardly heard of. The island of Ceylon, there is evidence to prove, has been freed from the small-pox by the introduction of vaccina. The small-pox was so prevalent in that island, that particular physicians were employed upon that disease only: it appears that those physicians are now compelled to seek another trade. It seems a paradox that the failures in vaccination should contribute to establish its prophylactic character. But if it were not a security generally, instead of hearing sometimes of a case of small-pox after vaccination, hundreds would occur. As far as the evidence of the private practice of individuals goes, ours has been decidedly in favour of vaccina. It may not be improper to state, that, in August last, a family of five children, who had been vaccinated at various previous periods from six years to one year, was inoculated with variolous virus, in its most active state, by the writer of this article; and that every individual was perfectly incapable of receiving small-pox.

In the long catalogue of diseases to which the whole of animated nature is subjected, fever, possibly, is the most destructive of life. In this sentiment we necessarily refer, however, to the term in its most enlarged meaning, comprehending the specific contagions by which the exanthemata are propagated; the infectious fever of camps, hospitals, and prisons, under the sweeping, and, perhaps, ill-applied term, *typhus;* the frequent and destructive form of fever referred to an external cause, unconnected with contagion or infection, but compounded, in various proportions, of many pernicious principles; as extreme fatigue, extraordinary privations, cold, improper food, and certain undefined effluvia (or if defined, still requiring that explanation which conveys a determinate idea) arising from swampy lands, and distinguished by the name *marsh miasmata.*

We have had the good fortune to see *variola*, the most destructive species of the contagious genus, if not deprived of its virulence, so shorn of its powers by the *vaccina*, as no longer to spread terror and dismay. Another species of this genus, the *scarlatina anginosa*, which has, though within a more confined range, been no less fatal than *variola*, we are assured, on very clear evidence, has been treated most successfully by a method, which, from former records of the disease, was esteemed capable of adding to its fatality. To Mr. Hamilton, of Ipswich, the profession owes this bold, but successful, deviation from an established practice. In the twenty-fifth volume of this Journal, page 101, will be found a detail of his practice, with the reasons upon which it was founded, and instances of its successful result. Since that period, the month of February 1811, we have heard of many additional instances of the happy effects of bleeding, the cold affusion, and sponging, in this formidable disease: we refer particularly to a letter by *Senex*, in the Journal for April 1811, page 298, and to cases of this disease by *Mr. Machell*, in the number for December, page 442.

Though we are neither the first to adopt new opinions and new practices, nor the last to leave the old, we must acknowledge the influence of the evidence before us, when contrasted with the fatality accompanying the opposite mode of treatment. Of such importance is this change in the treatment of *scarlatina anginosa*, that we do not hesitate to say every practitioner is called by the voice of science and humanity, to inquire into and make known facts respecting it. If this depleting practice * is ever right, there is no question that it must be safest and most effectual when employed early in the disease: but to what extent it may be carried, both as to time and degree, must be ascertained by experience and observation: and whether tonics and stimulants are admissible while increased action and increased temperature continue in the system, must be determined by the same means.

* The term *depletion* has generally, if not always, been employed, we believe, to express the emptying the system, by bleeding and purging; the accumulation of heat in fever has however been too generally overlooked: the abstraction of heat, or the keeping down the temperature in fever, is an object of the first consideration; and is, if we appropriate it justly, an indispensible part of the depleting practice.

 The

The attention of the medical faculty has of late been solicited to two distinct forms of fever, as far at least as names make distinctions,—the yellow-fever of the West Indies and America, and the remittent of Walcheren; both, perhaps, arising from some modification and combination of the causes productive of the febrile state, but unconnected with contagion or infection. The publications of Drs. Bancroft and Wright induce, at this time, a notice of these diseases. The discordance of opinions upon the cause of yellow-fever, is well known: and in a former Report we endeavored to state the evidence on the question of its infectious or non-infectious quality; when it appeared, though the contending parties agreed on some important facts in the natural history and treatment of the disease, that the evidence on this point was so balanced as to leave the question undetermined. A preponderance is given, however, to the opinion of its being non-infectious, by the greater number of practitioners who have seen the disease in those countries where its ravages have been made, supporting this conclusion: while the infectious quality of the fever is contended for by those who have never seen it, but have received the data upon which their belief is founded from written documents, or have been influenced by general analogies *.

Among those who have their knowledge of this disease from nature is Dr. Bancroft. When physician to the army, he had many opportunities of seeing this fever, and these have convinced him that it is not infectious †. If the

* Though this statement is generally true, yet it is fair to observe there are exceptions to it. Dr. Blane is one, and Dr. Chisholm, (Letter to Dr. Haygarth,) whose opportunities of seeing the disease and habits of investigation, entitle him to much credit, is another.

† "One fact which decidedly proves the yellow-fever to be destitute of any contagious power, is that of its never having been communicated to others by any one of the many thousands, who, in the West Indies, as well as at Charleston, Norfolk, Baltimore, Philadelphia, New York, &c. were removed beyond the *reach of marsh miasmata*, whilst laboring under the disease, or after having imbibed the poison; though, in many of these, the disease appeared in its worst forms, and proved mortal." Bancroft. Essay on Yellow-Fever, &c. 413. Dr. Bancroft takes the observations of many practitioners in support of this opinion.

typhus

*typhus icteroides* is not the product of animal contagion or infection, from whence does it arise? From *marsh miasmata* is the answer. This opinion, which brings the yellow-fever so near the Walcheren remittent, is supported by Dr. Bancroft; yet of *marsh miasmata* we know nothing but their effects, remittent and intermittent fevers. The nature and qualities of their constituent principles yet remain unknown; nay, it is even doubtful where these deleterious effluvia are produced in greatest abundance. "I ought here to notice," says Dr. Bancroft (208) "the well-known influence of clay, in promoting the formation of *marsh miasmata*, either as a stratum for the soil, in which they are formed, or when mixed with it in a large proportion." In another place he observes, "that the inhabitants of peat-bogs or moors are exempt from those intermittent fevers which are so frequent over marshy districts." We confess that our observations, made indeed in England only, have led to conclusions in direct opposition to those made by Dr. Bancroft.

In the extensive meadows and marshy lands lying in the course of the rivers of this country, formed generally of clay, both surface and substratum, subject to be inundated in summer and autumn, as well as winter, the remittent or intermittent fever is of rare occurrence. But in other districts, composed of a surface of peat for many miles together, with a substratum when of clay from four to ten feet below that surface, both remittents and intermittents are endemic, and in some seasons epidemic to an alarming extent. There may, however, have been some very material circumstance overlooked in forming our conclusions: and as the primary object is with us, (and we trust with all those who consider medicine to be a liberal and useful science,) to elicit truth, we cannot but indulge the hope that some of our numerous correspondents will furnish facts illustrative of these opposing conclusions. The meadow and marshy lands through which the Thames, the Severn, and other large rivers flow, and the fens of Cambridgeshire, Essex, Lincoln, &c. are the localities which will afford the materials to elucidate this question.

As

As the object of this Report is to state plainly such facts, arising from time to time in the science of medicine, and the auxiliary branches of natural history and philosophy, as may be deemed of sufficient importance, we touch but slightly on the different kinds of books; and this must be our apology to Dr. Wright, to whom we shall on a future occasion be more attentive.

On points more strictly theoretical, it would be an injustice to overlook the continuation and conclusion of Mr. Smith's "*Theory of Sensation.*" In a former Report (No 5.) we spoke of this essay, then in its commencement, with approbation. In its progress nothing has arisen to lower our opinion. As a specimen of well-devised experiments, perspicuous details, close reasoning, and ingenious induction, we have seldom seen any thing superior, on the difficult subject of the animal œconomy. The author has also interwoven practical observations, particularly on the cold affusion, not only important in themselves, but happily illustrative of his hypothesis.

Among the novelties of this period, we cannot leave unnoticed the "*Observations sur la physiognomie propre a quelques maladies chroniques,*" by Dumas, of Montpellier. The speculative disposition of this physician has always given to his productions an interest of a peculiar cast; but, though constantly ingenious, they have, generally, a coloring from imagination not perfectly according with a cautious investigation of facts, and a philosophic inquiry for truth. This we conceived to be remarkably conspicuous in his essay on the property which the different organs of the human frame have of being transformed into each other. In the instance before us we are, however, willing to believe that, as the conclusions are deduced more immediately from palpable appearances, and as the foundation of the principle lies more on the surface, and the results being less dependant on the feelings and descriptions of the patient, it may approach to that decisive character which *should* mark the whole of pathology.

According to M. Dumas, the physiognomic marks of phthisis pulmonalis are, a shining brightness of the eye, with with a prominence and red tinge of the eye-ball, a lengthening of the line between the orbits, a hollowness of the temples, and a sinking of the cheeks. A wan and waxen hue of the countenance, a languor in the eyes, a tarnished whiteness of the cornea, with a bloated distention of the skin, are the principal traits of the hydropic physiognomy, which is strongly marked in the pictures of Gerard Dow.

In hydrocephalus, the eye is thrust forward from the orbit, and half of it is hid under the eye-lid, giving a decided character to the face of those laboring under this disease. The visage seems enlarged, dilated, and altered from its natural shape, in venereal affections: a livid hue, faded eyes, discolored and hardened skin, give an expression of languor and dejection to the syphilitic physiognomy.

The scrophulous diathesis is marked by an enlarged head, particularly about the occiput; by prominent cheek bones; by a full and bloated visage; by a projecting squareness of the lower jaw, and an extended outline of the chin. Large lips, thickened eye-lids, wild, melancholy, and sometimes haggard eyes, the cornea of which have a bluish transparency, and their pupils dilated, fill up, as M. Dumas says, the strumous physiognomy.

To the arthritic physiognomy, a character, fixed and determined by certain rules, cannot be assigned: but it is considered by this ingenious physiologist, to have the greatest affinity with that expression of eye indicating a dull internal pain, and an air of languor and debility, which appears in the female countenance about the period of menstruation. The characteristic signs of some chronic complaints are asserted to be marked with such precision, as to determine with great exactness the nature of the disease. A chronic diarrhœa, a long-continued dysentery, obstructions of the liver and abdominal viscera, scirrhus and ulcer of the uterus, cancer, and general cachexia, are examples.

There is a permanent character arising out of the anatomical state of the parts, which determine the particular physiognomy of nervous diseases, and which is best marked when the specific cause arises from malformation. M. Dumas speaks of the following as permanent in many morbid states, but which which cannot, however, be said all of them to arise from organic defects. An effect resulting from a combination of pallid, dark, and yellowish tints, proper to the melancholic temperament, give to the physiognomy a character of sadness and suffering, finely expressed in the Antinous of the Capitol.

Lavater has given the portraits of two hypochondriacs, whose appearance was so changed by their disease, that they were hardly recognised as the same persons. Their eyes were sunken and haggard, and their whole physiognomy expressing that painful constraint which oppresses the vital functions in hypochondriasis. Mania presents a different expression according to the species of the disease: but it is always characterised by a combination of determinate traits. The researches of physicians have decided that there are specific marks characterising the different kinds of mental alienation.

The general character of the maniacal physiognomy consists in disordered and irregular lines, having an analogy with incoherence of ideas. Idiotism, however, shews itself rather by a regularity of visage, but cold, motionless, and inanimate; the face generally being of a proportion too large for the cranium. In one species of intellectual imbecility, that arising from hydrocephalus, and where the faculties of the mind seem totally obliterated, idiotism is connected with an enlarged cranium, evidently made so at the expence of the face*.

The physiognomic character of epileptics, more particularly drew the attention of Dumas. An examination of many persons suffering under this disease, enabled him, from actual admeasurements, to point out the peculiarities in the shape of the head which decide the epileptic physiognomy. A disposition in the muscles of the face to convulsive motions; lowering eye-brows, palpebræ nearly closed; projecting, fixed, shining eyes, with their pupils

* The brain in hydrocephalus will be in a state of compression, notwithstanding the increased volume of the cranium, from the accumulation of fluid, as much as if the boney structure had too small a dimension.

turned in opposite directions, give the obvious marks of this physiognomy. But, above all, he says, a peculiar facial angle distinctly impresses a character on the general physiognomy of the constitutional epileptic, or those whose disease arises from some organic defect in the brain. This peculiarity is a lowered proportion of the facial angle, which is always observed to be under 80 degrees. It is remarked, that there is a singular affinity between the facial angle of the greater number of epileptics and that of the negro, each receding from 80, and approaching to 70, degrees.

In 1804 and 1805, M. Dumas measured the facial angle of many epileptics in the hospital at Toulouse: four among these had it from 70° to 71°; three from 71° to 72°; and in one it was found below 70°. It is a practical fact of importance, and which we hope will be confirmed by additional observation, that, in those cases of epilepsy which are curable, the facial angle always approaches to 80°; and in those which arise from organic defect in the brain, and are consequently incurable, the facial angle is lowered to 72°; and even, in some instances, below 70°.

We are not convinced that the principle of M. Dumas may not be applied to many acute diseases; or that diseases, generally, do not give a distinct character of countenance, or, as this physician expresses it, a physiognomy peculiar to each. If the violence and termination of morbid affections are, from this source, occasionally obvious to superficial observers, what may not be expected when persons of talent, capable of discriminating and selecting, apply earnestly to the subject? The pencil will afford an auxiliary of immense importance; and, as a faithful and characteristic portrait, will, probably, depend on a few lines happily caught; when these lines are well understood, much of the difficulty will be vanquished. No branch of medical science seems so perfectly untouched as this; nor so likely, when executed with sufficient precision, to bring the reward of REPUTATION.

*Prince's Street, Cavendish Square,* R.

*December* 31, 1811.

