# Corrigenda

**Published:** 1812-02

**Authors:** 


### *CORRIGENDA.*

Page 35, in nota, for appropriate read *appreciate*.

